# Multiple cutaneous metastases of amelanotic melanoma

**DOI:** 10.11604/pamj.2018.29.141.14489

**Published:** 2018-03-02

**Authors:** Priscila Vinhal Grupioni, Fred Bernardes Filho

**Affiliations:** 1 Emergency Department, Hospital Imaculada Conceição da Sociedade Portuguesa de Beneficência, Ribeirão Preto, São Paulo, Brazil; 2 Department of Oncology, Hospital Imaculada Conceição da Sociedade Portuguesa de Beneficência, Ribeirão Preto, São Paulo, Brazil; 3 Dermatology Division, Department of Medical Clinics, Ribeirão Preto Medical School, University of São Paulo, Ribeirão Preto, Brazil

**Keywords:** Melanoma, amelanotic melanoma, neoplasm metastasis

## Image in medicine

An 86-year-old woman was admitted at the emergency room with decreasing consciousness level and severe pain in the lower right limb. Her past medical history was pertinent for a history of hypertension, diabetes mellitus, congestive heart failure, and a dual chamber pacemaker insertion. Positive findings on physical examination included ulcerated tumor in the right leg with exudate drainage and multiple erythematous nodules in her right lower limb. Histopathology of the ulcerated tumor's edge and one erythematous nodule showed solid and infiltrative epithelioid neoplasia, frequent atypical mitoses, and areas of necrosis and ulceration. Immunohistochemical evaluation of both lesions described above showed strong staining for vimentin and S100 and negative staining for P63, HMB-45, melan A, 1A4 (Alpha-Smooth Muscle Actin Antibody), desmin, CD20, CD3, CD30, CD10, LCA, PAX-8, AE1/AE3 and CD56. Histopathological and immunohistochemical findings revealed a malignant mesenchymal neoplasia, with immunophenotypic aspects of amelanotic melanoma. Patients who have advanced metastatic melanoma often present with multiple cutaneous metastases. Progression of advanced locoregional disease usually causes pain and functional impairment in the affected region. Lesions can ulcerate, bleed, and increase in number and size. Palliative treatments are therefore necessary.

**Figure 1:**
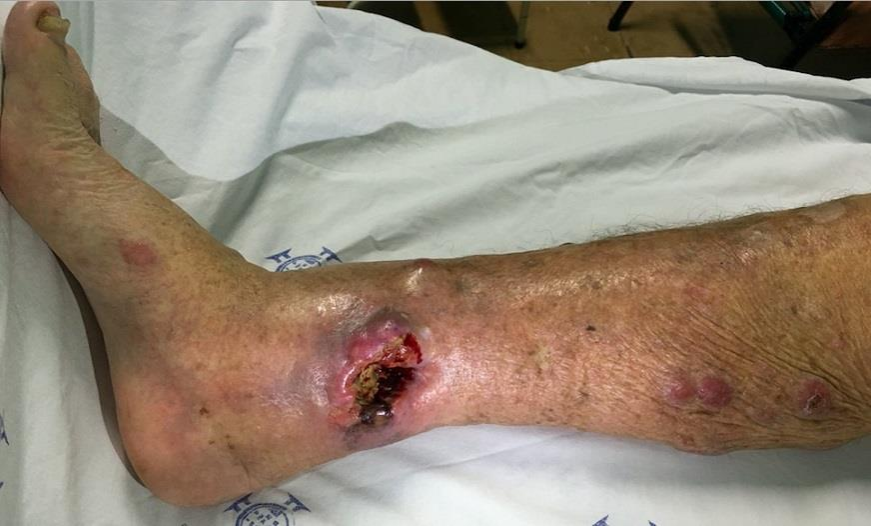
ulcerated amelanotic melanoma with multiple red cutaneous metastases

